# 

**Published:** 1832-04

**Authors:** 


### MONTHLY LIST OF MEDICAL BOOKS.

[*Medical Works cannot be entered on this List unless a copy be sent for the purpose, the titles of Books having frequently been sent to us as published which have not been published for weeks, or even months, after.*]

Remarks on the Necessity for establishing, in Liverpool, a Dispensary for Children. By Samuel Malins, m.d., Lecturer on Midwifery, &c.

(Part III. March 1832.) The Cyclopædia of Practical Medicine. Edited by John Forbes, m.d., A. Tweedie, m.d., and John Conolly, m.d.—Royal 8vo. pp. 112. Sherwood and Co. London.

A Letter to the Presidents of the Westminster Medical Society, on Cholera. By John Webster, m.d., Physician to St. George's and St. James's Dispensary.—London.

Dissertations on Malaria, Contagion, and Cholera; explaining the Principles which regulate Endemic, Epidemic, and Contagious Diseases, with a View to their Prevention: intended as a Guide to Magistrates, Clergymen, and Heads of Families. By Wm. Aiton, m.d. &c.—8vo. pp. 222. Longman and Co., London.

Dr. Townsend's Chart of the Physical Signs of Diseases of the Lungs.—John Taylor, London.

Published by Authority of the Central Board of Health. Report on the Chemical Pathology of the Malignant Cholera; containing Analyses of the Blood, Dejections, &c. of Patients labouring under that Disease in Newcastle and London, &c. By W. B. O'Shaughnessy, m.d.—8vo. pp. 72. Highley, London.

*** We have for some time looked anxiously for this report, and we regret extremely that it arrived at too late a period in the month to enable us to do it justice in the present number: in our next we shall give our readers a full account of its contents.

Lectures (delivered at the Mechanic's Institution) on Carbon, Oxygen, and Vitality, the three great Agents in the Physical Character of Man: with Remarks on Asiatic Cholera. By GEORGE REES, M.D. &c.—8vo. pp. 107. Highley, London.

Cholera, as it has recently appeared in the Towns of Newcastle and Gateshead; including Cases illustrative of its Physiology and Pathology, with a View to the Establishment of sound Principles of Practice. By T. M. GREENHOW, (of Newcastle upon Tyne,) Member of the Royal College of Surgeons in London, &c.—8vo. pp. 162. Highley, London.

Thoughts on Cholera Asphyxia. By ROBERT BREE, M.D., F.R.S., &c.—8vo. pp. 69. Wilson, London.

Anatomical Demonstrations or Colossal Illustrations of Human Anatomy. By Professor SEERIG. Translated from the German. Part II.—Schloss, London.

*** We have examined this work with more than ordinary care, as, from its cheapness, we were induced to fear it could not be very ably or correctly executed. We find, however, upon the strictest examination, that it possesses the unusual merit of being cheap in price and very valuable in its matter. The present part contains various illustrations of the Brain, the origin and passage of the Cerebral Nerves, &c. upon a "colossal" scale, which renders the study of the plates much more agreeable and improving than if they were of a smaller size. These "Anatomical Demonstrations" will be invaluable to students. We are called upon to express our admiration of the spirit which prompts Mr. Schloss to undertake the publication of so many useful works.

*** *Communications have been received from* Mr. MITCHELL, Dr. ROBERTS, Mr. G. T. BURNETT, *&c.*

